# Parental support in esports through the lens of the theory of planned behaviour

**DOI:** 10.3389/fspor.2024.1366122

**Published:** 2024-02-29

**Authors:** Joar Svensson, Oliver Leis, Michael G. Trotter

**Affiliations:** ^1^ School of Health and Medical Sciences, Örebro University, Örebro, Sweden; ^2^ Sport Psychology, Faculty of Sport Science, Leipzig University, Leipzig, Germany; ^3^ Department of Psychology, Umeå University, Umeå, Sweden

**Keywords:** esports, support, attitudes, structure, concern, physical activity

## Abstract

Esports have grown substantially in the last decade and may be an effective way of engaging and exposing the youth, who is not actively participating in traditional sports, to the benefits of sports related performance environments. However, due to negative stereotypes about gamers and concerns about esports, parents might be hesitant to support their children's esports participation and may instead actively discourage it. The purpose of this perspective article was to discuss the determinants of parental support based on the theory of planned behaviour. Parents attitudes seem to be mostly negative and their perceived behavioral control is likely low due to a lack of knowledge about esports. The subjective norms are mixed and seem to be growing progressively more positive. Based on the theory of planned behaviour, parents seem unlikely to support their children's esport participation, however, more research is needed. Recommendations on how to increase the likelihood of parental support are discussed.

## 1 Introduction

Electronic sports (esport) have grown substantially in popularity and market value over the last decade (1). Researchers recognize the potential of esports to attract young people [e.g., (2)] and cultivate a healthy lifestyle (3). Esports may be a new effective way to reach the approximately 20% of youth who are not engaged in the performance environments of traditional sports (4) and lead to many positive developmental outcomes [e.g., (5)]. For instance, esports programs have been effective at improving social-emotional and communication skills, leading to better social relationships outside the esports club [e.g., (6)]. Grassroots esports may also be an effective context to promote physical activity (PA) among youth, especially when aligned with a creative emphasis on play and enjoyment, rather than framing it as a mere “training” exercise (7). Although esports offers a unique and perhaps counterintuitive setting to foster healthy psychological development and health behaviour change (8), parents might be reluctant to support their children's esport involvement due to the negative stereotypes about video gamers [e.g., socially inept couch potatoes (9)] and negative beliefs about esports (10).

Parents play an important role in influencing and guiding youths’ involvement in structured activities (11) aimed at promoting positive adjustment and development (12). For example, in Nordic high school esports programs, the majority of parents (58%) whose children were involved either expressed no opinion, disagreed with, or actively discouraged their children's participation (13). Without parental support children may quit esports (14) or engage in unstructured play, lacking many essential components seen in organized sports activities including; supervision, guidance from adult leaders, rules, and structured practice and play [e.g., (15)]. The absence of these components has been associated with increased odds of health risk behaviors such as smoking, alcohol consumption and worsened academic achievement among youth (16). Parental support likely plays a pivotal role in children's esport participation, representing a relatively unexplored research area dependent on parental attitudes toward esports.

## 2 Theory of planned behaviour

**Figure 1:**
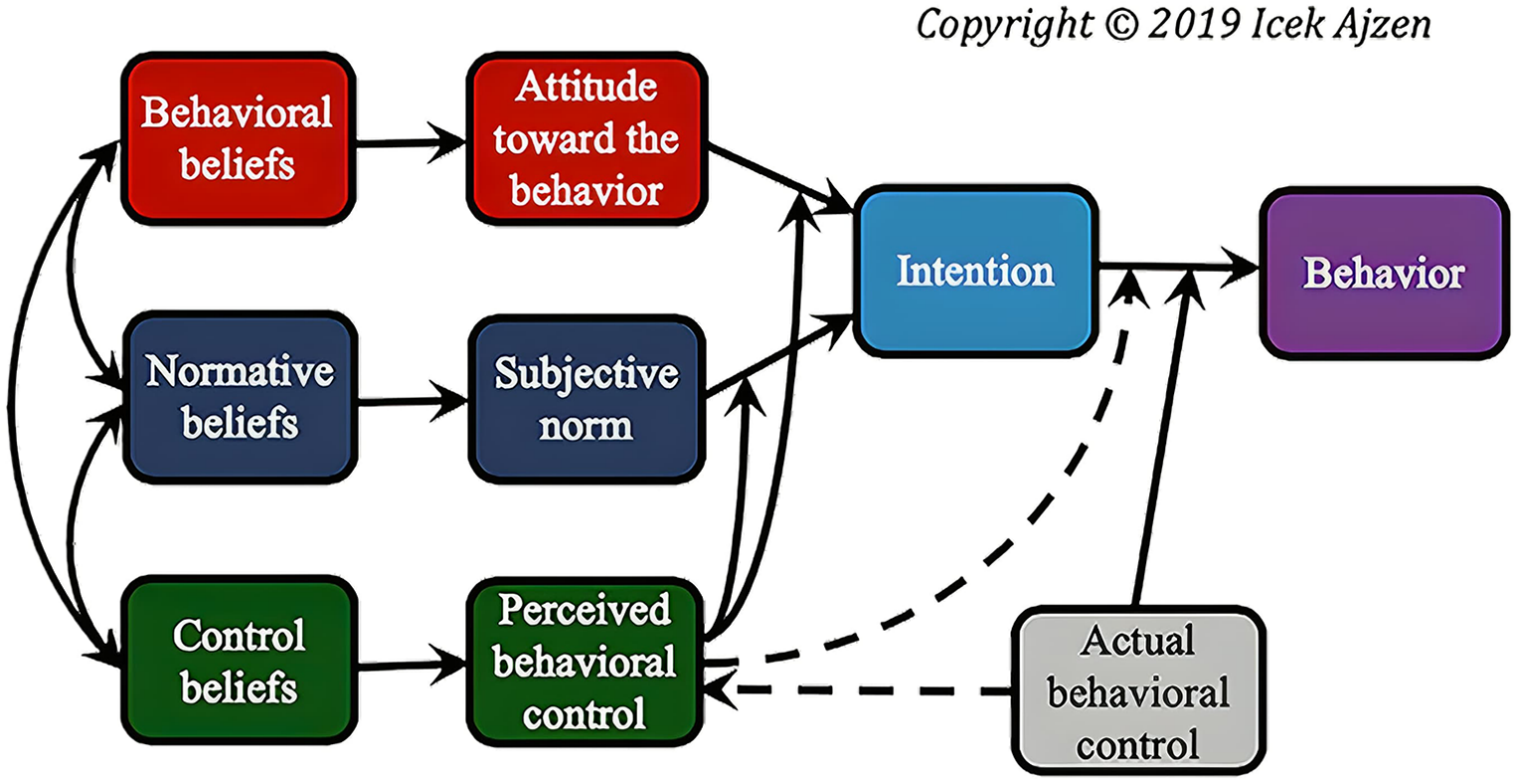
Theory of planned behaviour. From theory of planned behaviour by Ajzen (51) https://people.umass.edu/aizen/tpb.diag.html.

The theory of planned behaviour (TPB) is a framework that has been used to understand and predict behaviours (17). According to the TPB (18), an individual's behaviour is determined by the individual's intention, which is influenced by the determinants of intention; the individual's attitude, perceived behavioural control (PBC) and subjective norms (17). The subjective norm pertains to the opinion of a specific reference group (spouse, family, co-workers) regarding a behaviour, including whether they approve or disapprove of the behaviour and whether they themselves engage in it. Attitudes involve a mixture of the individual's beliefs about the experience of engaging in a specific behaviour and its outcome. Positive attitudes are more likely to strengthen an individual's intention to change their behaviour. PBC is an individual's ability to exert control over a behaviour. PBC may be influenced by factors such as previous experience and social support. The determinants of intention impact the individual's intentions towards the behaviour which in turn impacts the behaviour. The theory has been successful in explaining and predicting behaviours in a wide array of domains such as physical activity (17), esport (19) and smoking (20). TPB is grounded in the target behaviour, which is influenced by the strength of an individual's intention toward that behaviour. Furthermore, TPB posits that intentions are directly influenced by subjective norms, an individual's attitudes, and the degree of behavioural control (see Figure 1). However, individuals can be influenced by barriers such as limited time, financial constraints, inadequate skills, and a lack of resources, even if they have the intention to make a change. Furthermore, intentions toward a given behaviour are also impacted by subjective norms and personal attitudes. Subjective norms, attitudes, and PBC are based on anticipated outcomes rather than the actual outcomes. Moreover, attitudes toward a behaviour may change due to participation in the behaviour (17). Overall, the model has been used to predict and better understand behaviours in several domains including esports (10), exercise, and healthy diets (21).

## 3 Parents beliefs and attitudes towards esports

Studies have explored attitudes toward different aspects of esports such as participation, careers, and school competitions (10, 22). Regarding esport school competitions, Cho et al. (22) found that parents were resistant and bewildered towards the incorporation of esport in the classroom. Esport players had to negotiate with their parents to be allowed to participate in esports, often committing to dedicating time to homework to secure permission for participation (22). Similarly, a significant proportion of parents (58%) with children involved in high school esports in Scandinavia expressed either no opinion (44%), disagreement (7%), or actively discouraged (7%) their children's participation in high school esports programs (13). Wong et al. (10) supported this by highlighting that a considerable number of parents were hesitant and expressed negative attitudes towards their children's esports participation. This reluctance stemmed primarily from the belief that esports could detract time from academic studies. Esport players have themselves reported difficulties in balancing academics with esports (10). In addition, parents have discussed concerns about perceiving esports as insecure employment (23), a potential risk factor for video game addiction (24), and decreased mental and physical health (25). Another contributing factor to parents’ resistance might be the novelty of esports with parents being unfamiliar with esports and its distinction from video gaming (24). Due to these perceptions, many parents might be unsupportive toward their children pursuing esport careers (23). However, research has shown that the resistance from parents toward esport participation decreases when esport players become successful (10). Similarly, more positive media coverage of esports may erode parents negative attitudes and resistance to their children's participation (25). Although existing research provides insights into the extent of parental support for their children's esports participation (13) and the relationship between parental support and children's esports participation (10), there is a need for a more detailed understanding of the factors influencing these attitudes.

## 4 Subjective norms

Subjective norms regard different referent groups’ opinions about the behaviour. As the population of parents is broad, the following referent groups will be addressed briefly; the general public, the state, and sports organisations. Importantly, parents likely have other parents as a referent group but as their attitudes toward esport were previously discussed they will not be discussed in this section.

### 4.1 The general public

When discussing esports it is important to keep in mind that they are hardly distinguishable from video games to the general population (10), as such it is relevant to include video games. Madrigal-Pana et al. (26) found that older participants (75.4% of participants aged >50) displayed more negative attitudes toward video games compared to their younger counterparts (49.8% of participants aged 18–29). Common negative beliefs included the notion that video games cause addiction (75.5%) and negatively affect health (51.3%). However, participants also held positive beliefs, such as video games stimulating mental abilities (59.6%), providing relaxation from daily life (55.2%), and communication within a family environment (49.4%) (26). Given that concerns about video game addiction and health are significant issues related to esports, targeting these beliefs could potentially contribute to more positive subjective norms.

### 4.2 The state and sports organisations

There has been a growing number of countries recognizing esports as a sport, which could legitimize esports. For example, in 2003 only South Korea, China and Russia recognized esport as a sport. In 2023, over 14 countries recognized esport as sports [e.g., (27, 28)]. In 2021, Jenny et al. (29) reported that 74 higher institutions worldwide offer esport-related degrees ranging from courses to bachelor's and master's degrees in areas such as business/management and media/communication. Some countries such as the USA have started offering scholarships for esport players (30). Other countries have put finances towards health promotion initiatives in esports (7). The Danish government has appointed an esport panel to make recommendations about topics like talent development, sustainability, exercise, laws and rules (31). The state's position on esport seems to differ largely depending on the country. Parents in different countries might therefore be more or less positive towards esports.

Similarly, sports organisations have given esports increased recognition. Large organisations such as NHL (32), NBA (33) and FIFA (34) have organized esport leagues and championships. Esports have also been incorporated into prestigious events such as the Asian Games (35), and the Olympic Games via the Olympic Esport series (36). The acceptance of esports from large sports organisations is important as it helps legitimize esport within society and may be an influential subjective norm.

## 5 Perceived behavioural control

Multiple factors can influence PBC such as skills, ability, time, and money (17). Regarding skill and ability, esports is a new industry and parents may lack knowledge of esports (24). More specifically, parents might lack understanding of how esports are played, what esport practice entails (7) and time to support and guide their children through esports (23). Furthermore, parents also seem to lack interest in esports and might therefore not take the time to amend their lack of knowledge (7). As such, from a skill and ability perspective, parents may have a low PBC. Regarding the financial aspects, costs can be a barrier to sports participation (37). While this may apply to esports participation as well, a significant amount of players engage in esports. For example, the esport League of Legends has 180 million active players (38), while the esports Valorant and Counter-strike 2 have 22 million and 752 thousand active players respectively [(e.g., (39, 40)]. Although further research is needed to understand the effect of financial investments on esports participation, these numbers illustrate that many people have the necessary equipment. However, parents might lack knowledge about esport and may feel ill equipped to provide support (23). For example, esport players have reported frustration related to equipment issues (41). Without good knowledge in esports, parents will likely feel ill equipped to help solve the issues. Furthermore, esport players are less likely to seek parental support and advice due to their parents’ limited knowledge about esports (23). This reluctance may be amplified during adolescence, a phase when individuals typically seek greater independence (42). If the children are unwilling to receive parental support, it might also be difficult for parents to provide support. In summary, parents may feel high PBC concerning financial and time aspects, but lower PBC related to their ability and knowledge of esports and their children's resistance toward support.

## 6 Practical recommendations

Based on the determinants of behaviour (i.e., attitudes, social norms and PBC parents seem unlikely to support their children's esport participation. If attitudes toward esports and parents PBC became more positive the likelihood of parental support could be increased. Attitudes toward esport seem to be mostly concerned with health aspects (addiction and physical health), academic success, and future careers. It is unclear if esport players meet the physical activity recommendations as researchers have found that that esport players both meet [e.g., (43, 44)] and do not meet the guidelines [(e.g., (45, 46)]. Regardless, if esport players meet the physical activity recommendations or not, parents could play an active role in their children's esport participation and provide a structure that incorporates physical activity, thereby helping their children meet the physical activity guidelines whilst getting the benefits of structured activities.

Regarding academic success parents are concerned that esports might distract esport players from studying (23). This concern has been echoed by esport players, discussing the difficulties of balancing academics with esports [e.g., (10)]. Another concern from parents relates to the future career prospects associated with becoming a professional esport player (23). Esports are highly competitive (14) and the average career span is relatively short (47). There are, however, several esports-related jobs that players could pursue if they fail to become professional players (29), including esport coach, broadcaster and HR manager [for an extensive list of esport related jobs see Scott et al. (48)]. Esport players also develop transferable skills that could help them in other jobs (49). Furthermore, parental support and the provision of structure could help esport players better balance esports with academics, which could provide a backup plan if their esports pursuits fail.

Regarding PBC, parents seem to be lacking in esports knowledge (23) which could hinder them from supporting their children even if they wanted to. Parents who lack knowledge in esports would likely benefit from receiving information and guidelines from fields such as sport psychology to help them better structure their children's esport participation (5). For example, information from sport psychology could aid parents in developing their children's ability to develop a growth mindset, set appropriate goals and cope with harassment which have been detailed as important mental skills for esport players [e.g., (50)]. Initiatives such as the Danish initiative to incorporate esport into traditional sports clubs (7) could also be helpful as they remove competence requirements on the parents.

## 7 Future research

Given the limited research on parents’ attitudes toward esports, a primary focus should be placed on gaining a better understanding of this aspect. Given that esports have been recognized as an avenue for physical and psychological development of young individuals (8), it is important to highlight the need for further investigation into concerns about potential negative impacts of youth esports (4, 24). Furthermore, attitudes toward esport could differ between different countries and cultural contexts. As such, it could be interesting to explore parental support based on a TPB perspective from different countries and cultural contexts. Future research could also explore which determinant of behaviour (i.e., attitudes, subjective norm and PBC) is most impactful in improving parental support. Another important aspect of research involves investigating where negative attitudes and public perceptions of esports diverge from reality (9). This research is needed for esports clubs and school programs to effectively educate the public about the potential health promotion role that esports can fulfill.

## 8 Conclusion

Esports, especially structured esports have many potential benefits, but parents might be hesitant to support their children's esport participation. Lack of parental support places children at a higher risk of missing out on positive outcomes associated with esports and structured activities, making them more prone to experiencing potential negative consequences. Parental support is determined by their attitudes, PBC, and social norms (51). Parents’ social norms appear varied, but parental attitudes and PBC predominantly lean toward the negative spectrum. The negative attitudes revolve around concerns about their children's health and academic success, while the lack of PBC is based on a lack of knowledge about esports. Enhanced positive exposure to esports and the provision of guidelines for children's esports participation could contribute to more positive parental attitudes and improved esports competence. This, in turn, may increase the likelihood of parental support, allowing children to derive the benefits of structured activities.
